# When Autonomy Fails: Ethics, Philosophy, and the Legal Duty of Palliative Care - Reflections on End-of-Life Medicine in the 21st Century

**DOI:** 10.1017/S1478951525101028

**Published:** 2025-11-04

**Authors:** João Carlos Geber-Júnior

**Affiliations:** 1 Center for Oncologic Intercurrence Care (CAIO), Cancer Institute of the State of São Paulo (ICESP), Hospital das Clínicas, Faculty of Medicine, University of São Paulo (FMUSP), São Paulo, Brazil; 2 Faculty of Health Sciences, São Paulo, Brazil; 3 Hospital Israelita Albert Einstein, São Paulo, Brazil

Modern medicine has achieved remarkable success in extending human life through technological innovation and aggressive clinical interventions. Yet this very success has created an ethical paradox: we are increasingly able to prolong life, but often unable to address the suffering, meaning, and dignity of those who are dying. In this context, palliative care emerges not merely as a subspecialty, but as an ethical reorientation of medicine itself – an approach that demands philosophical and moral reflection (Beauchamp and Childress 2019; Cassell 2004; Saunders 2003).

The origins of modern palliative care are closely associated with Cicely Saunders and the hospice movement, which offered a moral critique of a healthcare system that prioritized cure over comfort and intervention over presence (Clark 2018; Saunders 2003). Her concept of “total pain” integrated physical, psychological, social, and spiritual suffering. This became a pivotal moment in medical ethics: a call for medicine to care not only for diseases, but for persons in their wholeness (Cassell 2004).

The four foundational principles of biomedical ethics – autonomy, beneficence, non-maleficence, and justice – continue to shape end-of-life decision-making (Beauchamp and Childress 2019). Yet palliative care reveals the limits of these principles, especially the ideal of rational autonomy. Near death, many patients experience cognitive decline, dependency, and emotional distress that compromise their capacity for independent decision-making. In such moments, ethical reasoning must move beyond individual choice and incorporate vulnerability, relationality, and context (Carel 2016; Jonas 1984; Ricoeur 1992).

The prevailing model of autonomy in clinical ethics is influenced by Harry Frankfurt, who conceived of persons as beings capable of reflecting on their desires – second-order volitions (Frankfurt 1971). This framework assumes stability, rationality, and deliberation. Yet in serious illness, patients may be confused, in pain, or emotionally overwhelmed. Their needs often exceed the procedural scope of advance directives or verbal consent (Carel 2016; Cassell 2004). The patient is no longer only a decision-maker, but a sufferer requiring ethical presence and interpretive care.

Philosopher Paul Ricoeur offers an alternative: ethics grounded in the narrative identity of a person, which unfolds over time and in relation to others (Ricoeur 1992). In his view, autonomy is never absolute – it is always in dialogue with solicitude, the mutual care that binds us. Similarly, Havi Carel’s phenomenology of illness shows how serious disease can rupture one’s sense of body, time, and identity, demanding not decisions but listening and companionship (Carel 2016). Hans Jonas adds a compelling vision of ethical responsibility, especially toward those who cannot advocate for themselves, such as the frail or unconscious (Jonas 1984). Emmanuel Levinas deepens this stance by locating ethics in the face-to-face encounter with the other – an obligation that precedes will or reason (Levinas 1969).

These philosophical approaches have concrete legal implications. In Brazil, Resolution CFM nº 1.995/2012 recognizes the legal validity of advance directives and the patient’s right to refuse disproportionate treatment, even when terminally ill or incapacitated (Conselho Federal de Medicina 2012). Internationally, the Oviedo Convention affirms the primacy of human dignity and limits on futile interventions (Council of Europe 1997). Brazil’s Constitution further enshrines the dignity of the human person as a foundational right (Brasil 1988). These documents align with the moral rationale behind palliative care as a duty – not a concession.

Nevertheless, structural and cultural barriers persist. Fear of litigation, emotional pressure from families, and the normalization of aggressive care often result in the prolongation of suffering rather than the protection of dignity (Dallari and Garrafa 2020; Dantas et al. 2021). A 2019 study in *The Lancet Global Health* projected that the number of people experiencing serious health-related suffering will double by 2060, especially in low- and middle-income countries, where access to palliative care is limited (Sleeman et al. 2019).

Another crucial obstacle is medical education. Many professionals receive extensive training in diagnosis and intervention, but little in narrative listening, ethical reasoning, or presence in the face of death (Charon 2006; Moreira and Zoboli 2020). This disconnect leaves clinicians technically prepared, but ethically disoriented when cure is no longer possible.

A cultural transformation is thus essential. Palliative care must be seen not as a secondary option, but as a clinical and ethical standard – an affirmation of medicine’s commitment to relieve suffering when healing is no longer an option (Cassell 2004). Dignity, in this light, is not defined by autonomy or functionality, but by how we respond to vulnerability with compassion and responsibility.

Ultimately, palliative care is more than a clinical practice. It is a moral stance – a commitment to accompany, to witness, and to honor the human experience at its most fragile. Far from signaling medical failure, to care well for someone at the end of life may be the most radical act of medicine.
